# Conservative Surgical Management for Pulmonary Hydatid Cyst: Analysis and Outcome of 148 Cases

**DOI:** 10.1155/2016/8473070

**Published:** 2016-08-24

**Authors:** Mohammed Aldahmashi, Mohamed Alassal, Ibrahim Kasb, Hany Elrakhawy

**Affiliations:** ^1^Cardiothoracic Surgery Department, Thamar University, Thamar, Yemen; ^2^Prince Abdullah Bin Abdulaziz Bin Musaed Cardiac Center (PAAMCC), Arar, Saudi Arabia; ^3^Cardiothoracic Surgery Department, Benha University, Benha, Egypt; ^4^King Salman Heart Center (KSHC), King Fahd Medical City (KFMC), Riyadh, Saudi Arabia; ^5^Nasser Institute for Research, Cairo, Egypt

## Abstract

*Background*. Hydatid cyst (HC) disease is endemic in many developing countries, like Yemen, Egypt, and Saudi Arabia, especially in the rural regions. The disease has a variable clinical courses and even might be asymptomatic for many years.* Objectives*. In giant and large pulmonary hydatid cysts, pulmonary resection is the usual method of surgical treatment. In this study, we aimed to evaluate the lung conservative surgery in treatment of cases with giant and large hydatid lung cysts, as an effective method of management.* Patients and Methods*. Between January 2009 and August 2014, a total of 148 patients with pulmonary hydatid cysts were operated and their data was reviewed retrospectively and analyzed. Out of these cases, 52 (35.14%) cysts with more than 10 cm in diameter and 36 (24.32%) cysts with 5–9 cm were regarded as giant and large hydatid lung cysts, respectively. The small cysts less than 5 cm were presented in 8 (5.4%) cases only; other cases had ruptured cysts. Preservation of the lung tissues during surgery by cystotomy and Capitonnage was our conservative surgical methods of choice.* Results*. Eight patients developed bronchopleural fistula (BPF); of them, 4 BPFs have healed with chest tube and physiotherapy, but in the other 4 patients reoperation was done for the closure of persistent BPF. No mortality was observed in the present study.* Conclusion*. We conclude that conservative surgical procedure can achieve complete removal of the pulmonary hydatid cyst. Enucleation of the intact huge cysts is safe. Careful and secured closure of the bronchial communication should be done by purse string or figure-of-8 sutures, with or without Teflon pledgets. These simple procedures are safe, reliable, and successful.

## 1. Introduction

Hydatid disease has been known since Hippocrates; it is a parasitosis caused by* Echinococcus granulosus* which accidently infect human with no rule in its life cycle; canines as dogs are the definitive host, while intermediate hosts are castles and sheep. Echinococcosis remains an endemic surgical problem in countries where sheep and cattle raising is carried out, particularly in many Middle East developing countries [1–3].

The disease has a variable clinical course. Hydatidosis may be asymptomatic for many years. It may become evident when a cystic lesion is noted during imaging for other reasons. It may also be symptomatic depending on the size, location, and complications of the cyst [4].

Radiologically, intact hydatid cyst of the lung has regular outline, and ruptured cysts may appear as blurred shadow and can be mistaken for carcinoma or tubercular focus. Impending rupture of the hydatid cyst can appear radiologically as crescent sign, inverse crescent sign, water lily, or camalote sign, which is due to collapse of endocyst and partial evacuation of its fluid [3, 5].

Laboratory diagnosis of hydatid disease includes Casoni's intradermal test or serologically ELISA for detection of immunoglobulins G, E, and M, The detection of IgG antibody is more sensitive and specific for diagnosis of human hydatidosis [6].

Surgical treatment is preferred in hydatid cysts of the lung [7–9]. Cystotomy and Capitonnage are the conservative surgical methods of choice as they preserve lung tissues [10].

## 2. Patients and Methods

Our study is a retrospective observational study done on patients from 3 tertiary centers in Middle East: Egypt, Saudi Arabia, and Yemen. After approval of the study protocol by the Local Ethical Committee, 148 cases between January 2009 and August 2014 of pulmonary hydatid cyst disease were reviewed retrospectively. In 52 of these cases, cysts were of 10 cm or greater in dimension (Figure 5) and were rated as giant or huge cysts, in 36 cases the hydatid cysts were less than 9 cm. The age, gender, symptoms, image findings of the cyst (dimensions, ruptured or nonruptured), operative procedures, complications, and hospital stay of the patients were obtained from charts.

**Figure 1:**
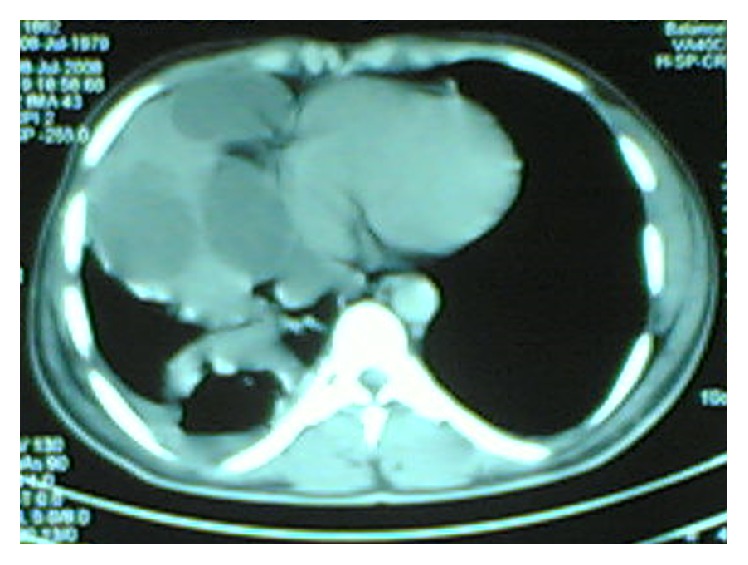
CT chest showing multiple large pulmonary hydatid cysts.

**Figure 2:**
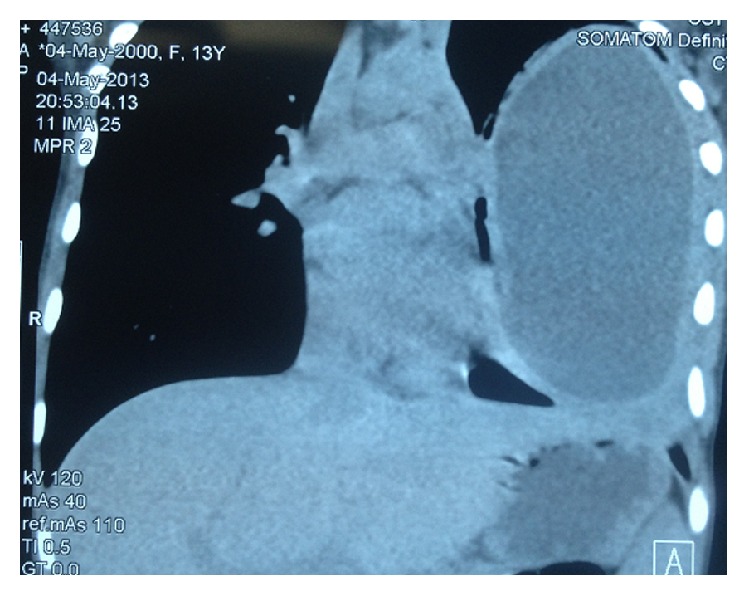
CT chest showing giant single pulmonary hydatid cyst.

**Figure 3:**
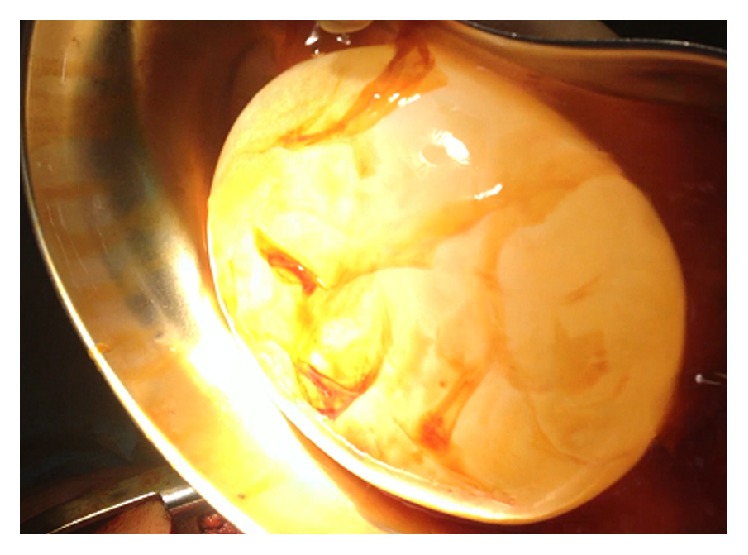
Photo showing large hydatid cyst after surgical excision (enucleation).

Preoperative evaluation was done by means of physical examination and laboratory investigations; in addition, specific anti-*Echinococcus* IgG were also performed. Radiological diagnosis was achieved by chest X-ray and computed tomography (CT) scan of the chest and the upper abdomen. A diagnosis of complicated hydatid cyst was made based on chest X-ray, CT, and medical history of sudden coughing with expectoration of salty hydatid fluid or purulent (pus) sputum in infected HC (Figures 1, 2, and 3).

## 3. Operative Techniques

All procedures were performed under general anesthesia, with double-lumen endotracheal tube and single-lumen endotracheal tube for the few younger patients.

The surgical approach was posterolateral or lateral thoracotomy in 140 patients depending on the cyst location. In 8 patients with bilateral hydatid cysts, median sternotomy was the surgical approach in these patients. Right lateral thoracotomy with a transdiaphragmatic approach was performed in 4 patients with associated single huge liver cyst.

The surgical procedure of choice was cystotomy with Capitonnage. When the hydatid cyst was identified, the surgical wound and adjacent tissue were covered with packed gauges soaked in 10% povidone-iodine so that only the area of the lung containing the cyst was exposed. In patients with ruptured and/or infected complicated cysts, after removal of the germinative membrane, the cystic cavity was carefully cleaned by suction and irrigated with 10% povidone-iodine and hypertonic saline 2.7% and then reexamined for remnants of cystic contents. Cystectomy (excision of the entire intact cyst by enucleation using Barrette technique) and closure of bronchial openings were identified by irrigating hypertonic saline solution while the anesthesiologist inflates the lung. Closure was done by 3/0 PDS sutures and sometimes with Ethibond sutures with or without pledgets according to the bronchial opening size and the surrounding tissue, and then Capitonnage was performed to obliterate the cyst space. Decortication was performed in patients with pleural complications. Medical treatment in the form of Albendazole was given in a dose of 10 mg/kg/day for 6 months postoperatively to those having ruptured or multiple cysts, but for 3 months to those having intact cysts; medication for 28 days was followed by a 7-day pause. Liver function tests were checked at follow-up. Investigations were made to evaluate the parenchyma and lung functions, preoperatively and at one-year follow-up postoperatively (Tables 1 and 2).

## 4. Statistical Analysis

Data were collected and compared afterward. The information was entered in a computer-designed format to facilitate analysis by using the SPSS 9.0 statistical program for Windows. Morbidity of the ruptured cysts and the intact cysts was compared. Comparison for hospital stay of both groups was calculated using the Mann–Whitney* U* test. The significance of the differences was calculated by the Wilcoxon test for the paired groupings. Analysis was carried out by the *χ* ^2^ tests for qualitative variables. In all cases the results were considered statistically significantly when *p* < 0.05.

## 5. Results

104 (70.27%) patients were male and 44 (29.73%) female with an average age of 27.75 years (range 7–56 years). 96 patient presented with intact cyst(s) and the others were ruptured cysts. In our practice, conservative surgical techniques, such as cystectomy plus closure of bronchial openings and Capitonnage of the residual cystic space, constituted the routine surgical approach. For intact cysts, cystectomy (enucleation) was the most frequent applied operative procedure; out of them only 4 were ruptured during enucleation procedure.

For the ruptured HC (37.84%, *n* = 56, noting that 4 cases had both intact and ruptured pulmonary hydatid cysts) the techniques were dependent on the complications; decortications were done in 32 cases (21.62% of the total number of cases) ande cystectomy (removal of remnants of germinative membranes and laminated membranes) and Capitonnage were done in 24 cases (16.22%).

Postoperative complications were infrequent and no mortality was seen. Prolonged parenchymal air leak (>5 days) was observed in twelve patients; eight of them had BPF. Air leak was managed in eight of them by chest physiotherapy and chest tube drainage. Rethoracotomy for BPF repair and closure was done for four cases. Atelectasis developed in 4 cases and resolved after few days of aggressive chest physiotherapy.

Albendazole was given to all patients postoperatively. It was administered as 10 mg/kg/day in 2 divided doses with maximum of 400 mg twice daily; the treatment was undertaken for 3 cycles of 4 weeks with pause interval one week in patients with intact cyst and for 6 cycles in patients with complicated or multiple cysts. CBC and liver function tests were followed up for any derangement. No patient had elevated test results during the course. During follow-up of all patients for one-year period, no recurrences were seen on chest radiography (Table 3).

## 6. Discussion

The most common localization of hydatid cyst is the liver with 50–60% and secondly the lungs (10–30%) [11–13].

Hydatid cyst should receive treatment as soon as diagnosis is established, since it may cause serious complications by means of rupture into bronchi and pleural cavity or vital organ compression [14].

In our study, the presenting symptoms of hydatid cyst had a wide range starting from being asymptomatic and accidently discovered to massive hemoptysis; 10.8% of patients were asymptomatic; the most presenting symptom was cough in 73.6% of patients; other symptoms were dyspnea in 62.2%, chest pain 64.9%, and fever 14.9%; these symptoms were also reported in many studies for pulmonary hydatid [15–18].

**Figure 4:**
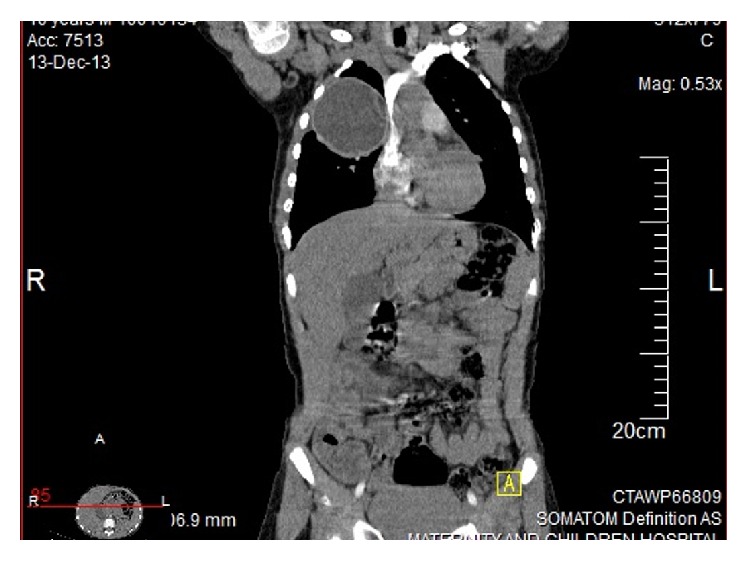
Large hydatid cyst compressing the SVC.

**Figure 5:**
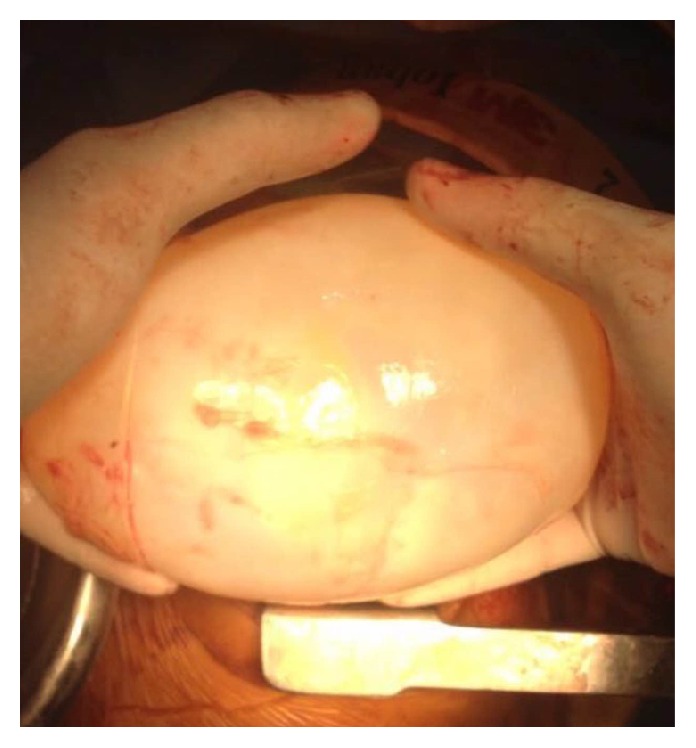
Giant hydatid cyst after enucleation.

The uncommon, but serious presentations in our subjects were in the form of positional hypotension and fainting that were reported in one of our cases due to superior vena cava compression (Figures 4 and 5).

Hemoptysis is not uncommon presentation of pulmonary hydatid disease [19, 20]. Massive hemoptysis (more than 600 mL/day) was the most serious presentation which was reported in 3 cases in our series; all of them were due to infected hydatid; mild to moderate hemoptysis was reported in 5 cases in our series.

Lung tissue should be preserved and resection should be avoided whatever the cyst size. Recurrence is very low. Although we had a parenchyma preserving approach, no recurrence was observed in our series; this was similar to another study [12]. Resection is not recommended unless whole lobe is destroyed. In our practice, conservative surgical techniques, such as cystectomy plus closure of bronchial openings and Capitonnage of the residual cystic space, constituted the routine surgical approach. In many cases in our study, a radio-opaque shadow appeared in chest X-ray and chest-CT after the Capitonnage; this shadow represents sutured lung tissues; it disappears within 3 weeks to 3 months.

The main aim of surgery in hydatid cysts is total excision [14]. Shields stated that lobectomy should be performed in cases where more than half of the lobe is involved [21]. Though unlikely, we did not do lung resection in our patients. In all patients, conservative surgical procedures were employed. Barrett and Thomas enucleated endocyst and obliterated cavity [22]. Burgos et al. did cystectomy in 312 out of 508 procedures for hydatid cysts of lungs [23]. Salih et al. observed that lung preserving surgery is the treatment of choice in hydatid lung in their study of 405 patients [24]. Others concluded that conservative procedure (enucleation with Capitonnage) remains the best treatment of simple hydatid cysts [25].

## 7. Conclusions

Parenchymal preservation by cystotomy with Capitonnage still remains a valid surgical method for hydatid cysts of the lungs, with excellent surgical results and fewer complications. Adding Albendazole postoperatively reduces the chance of recurrence to be almost nil.

## Figures and Tables

**Table 1:** Preoperative diagnosis of hydatid cyst.

Variable		Intact cyst (*n* = 96)		Complicated cyst (*n* = 52)		Total (*n* = 148)	
		Freq.	%	Freq.	%	Freq.	%
Chest X-ray	Yes	88	91.7%	52	100.0%	152	94.6%
	No	16	8.3%	0	0.0%	8	5.4%

Contrast enhanced CT chest	Yes	96	100.0%	52	100.0%	148	100.0%

IgG ELISA	Positive	88	91.7%	28	53.8%	116	78.4%
	Negative	0	0.0%	8	15.4%	8	5.4%
	Not done	8	8.3%	16	30.8%	24	16.2%

**Table 2:** Characteristics of the patients and the cyst.

Variable	Intact cyst (*n* = 96)		Ruptured cyst (*n* = 52)		Total (*n* = 148)		*p* value
	Freq.	%	Freq.	%	Freq.	%	
Age (year)							0.542
<20	24	25.0%	4	7.7%	28	18.9%	
20–30	40	41.7%	24	46.15%	64	43.2%	
>30	32	33.3%	24	46.15%	56	37.8%	
Gender							0.057
Male	56	58.3%	48	92.3%	104	70.3%	
Female	40	41.7%	4	7.7%	44	29.7%	
Complication at presentation							NA
Empyema			32	61.5%	32	21.6%	
Pneumothorax, pleural effusion			8	15.4%	8	5.4%	
Pneumothorax			8	15.4%	8	5.4%	
Tension, pneumothorax			4	7.7%	4	2.7%	
Cyst size							NA
<5	8	8.3%	0	0.0%	8	5.4%	
5–9	36	37.5%	16	30.8%	52	35.1%	
≥10	52	54.2%	36	69.2%	88	59.5%	
Multiplicity							0.538

*p* value is significant if <0.05.

**Table 3:** Outcomes of hydatid cyst surgery.

Variable	Enucleation (*n* = 96)		Cystotomy (*n* = 52)		Total (*n* = 148)		*p* value
	Freq.	%	Freq.	%	Freq.	%	
Complications							NA
Air leak	4	4.2%	8	15.4%	12	8.1%	
Atelectasis	1	1.04%	3	5.8%	4	2.7%	
Hospital stay (day)							
Median (IQR)	10.0 (2.0)		10.0 (2.0)		100 (2.0)		0.1
5 days	28	29.2%	20	38.5%	48	32.4%	
6 days	44	45.8%	20	38.5%	64	43.2%	
7 days	20	20.8%	8	15.4%	28	18.9%	
8 days	4	4.2%	4	7.7%	8	5.4%	
Reoperation for closure of BPF	0	0.0%	4	100.0%	4	2.7%	NA
Normal CXR—1–3-month F∖U	96	100.0%	52	100.0%	148	100.0%	NA
Recurrence—1-year F∖U	0	0.0%	0	0.0%	0	0.0%	NA
Mortality	0	0.0%	0	0.0%	0	0.0%	NA

NA: not applicable.

*p* value > 0.05 is insignificant.

## References

[1] Shalabi R. I., Ayed A. K., Amin M. (2002). 15 Years in surgical management of pulmonary hydatidosis. *Annals of Thoracic and Cardiovascular Surgery*.

[2] Kiliç D., Erdogan B., Habesoglu M. A., Hatipoglu A. (2003). Multiple primary chest wall hydatid cysts associated with spinal canal involvement. *Interactive Cardiovascular and Thoracic Surgery*.

[3] Kaur M., Singh R. (2013). Ruptured pulmonary hydatid cyst: the camalote sign. *Indian Journal of Clinical Practice*.

[4] Grossi G., Lastilla M. G., Teggi A. (1991). 420 patients with hydatid cyst: observations on the clinical picture. *Arch Hydatid*.

[5] Erdem C. Z., Erdem L. O. (2003). Radiological characteristics of pulmonary hydatid disease in children: less common radiological appearances. *European Journal of Radiology*.

[6] Tenguriaa R. K., Naika M. I., Bhata J. A., Fomdab B. A. (2013). Comparison of Casoni's intradermal test with enzyme linked immunosorbent assay in the diagnosis of human hydatid disease. *International Journal of Current Science E*.

[7] Erdoğan A., Ayten A., Demircan A. (2005). Methods of surgical therapy in pulmonary hydatid disease: is capitonnage advantageous?. *ANZ Journal of Surgery*.

[8] Halezaroglu S., Çelik M., Uysal A., Senol C., Keles M., Arman B. (1997). Giant hydatid cysts of the lung. *The Journal of Thoracic and Cardiovascular Surgery*.

[9] Karaoglanoglu N., Kürkçüoglu IC., Görgüner M., Eroglu A., Turkyilmaz A. (2001). Giant hydatid lung cysts. *European Journal of Cardio-Thoracic Surgery*.

[10] Yalçinkaya I., Er M., Özbay B., Uğraş S. (1999). Surgical treatment of hydatid cyst of the lung: review of 30 cases. *European Respiratory Journal*.

[11] Sadrizadeh A., Haghi S. Z., Masuom S. H. F., Bagheri R., Dalouee M. N. (2014). Evaluation of the effect of pulmonary hydatid cyst location on the surgical technique approaches. *Lung India*.

[12] Şehitoğulları A. (2007). Our results in surgical treatment of hydatid cyst of the lungs. *European Journal of General Medicine*.

[13] Ülkü R., Yılmaz H. G., Onat S., Özçelik C. (2006). Surgical treatment of pulmonary hydatid cysts: report of 139 cases. *International Surgery*.

[14] Ramos G., Orduña A., García-Yuste M. (2001). Hydatid cyst of the lung: diagnosis and treatment. *World Journal of Surgery*.

[15] Ekim H., Özbay B., Kurnaz M., Tuncer M., Ekim M. (2009). Management of complicated giant thoracic hydatid disease. *Medical Science Monitor*.

[16] Kuzucu A., Soysal Ö., Özgel M., Yologlu S. (2004). Complicated hydatid cysts of the lung: clinical and therapeutic issues. *Annals of Thoracic Surgery*.

[17] Mamishi S., Sagheb S., Pourakbari B. (2007). Hydatid disease in Iranian children. *Journal of Microbiology, Immunology and Infection*.

[18] Solak H., Yeniterzi M., Yüksek T., Anil N., Göktogan T., Ceran S. (1990). The hydatid cyst of the lung in children and results of surgical treatment. *Thoracic and Cardiovascular Surgeon*.

[19] Özsürekçi Y., Parlakay A. Ö., Cengiz A. B. (2013). Atypical presentation in hydatid disease: hemoptysis. *Türkiye Parazitoloji Dergisi*.

[20] Toleti S., Subbarao M., Dwarabu P. (2012). Hydatid disease of the lung presenting with hemoptysis and simulating a lung abscess. *Tropical Parasitology*.

[21] Rochan R. B., Rice C. L., Carrico C. L., Shields T. W. (2000). Hydatid disease of the lung. *General Thoracic Surgery*.

[22] Lichter I. (1972). Surgery of pulmonary hydatid cyst: the Barrett technique. *Thorax*.

[23] Burgos L., Baquerizo A., Muñoz W., de Aretxabala X., Solar C., Fonseca L. (1991). Experience in the surgical treatment of 331 patients with pulmonary hydatidosis. *The Journal of Thoracic and Cardiovascular Surgery*.

[24] Salih O. K., Topcuoğlu M. Ş., Çelik Ş. K., Ulus T., Tokcan A. (1998). Surgical treatment of hydatid cysts of the lung: analysis of 405 patients. *Canadian Journal of Surgery*.

[25] Wani N. A., Hamid M. B., Hassan M., Bhat G. H., Hussain A. M. S. (2005). A brief clinical study and management of lung cysts in Kashmir Valley. *JK-Practitioner*.

